# Validation of the comprehensive online sleep monitoring scale in a large population sample

**DOI:** 10.3389/fpsyg.2026.1840525

**Published:** 2026-08-18

**Authors:** Laila Rida, Konstantinos Ioannidis, Samuel R. Chamberlain, Jon E. Grant, Peter Hellyer, Valentina Giunchiglia, William Trender, Karla V. Allebrandt, Sukhi Shergill, Steven C. R. Williams, Adam Hampshire

**Affiliations:** 1 Department of Neuroimaging, Institute of Psychiatry, Psychology and Neuroscience, King’s College London, London, United Kingdom; 2 Department of Brain Sciences, Imperial College London, London, United Kingdom; 3 Department of Psychiatry, Faculty of Medicine, University of Southampton, Southampton, United Kingdom; 4 NHS Southern Gambling Service, Hampshire and Isle of Wight Healthcare NHS Foundation Trust, Southampton, United Kingdom; 5 Department of Psychiatry and Behavioral Neuroscience, University of Chicago, Chicago, IL, United States; 6 Department of Biomedical Informatics, Harvard University, Cambridge, MA, United States; 7 Department of Medicine, Boehringer Ingelheim International GmbH, Ingelheim am Rhein, Germany; 8 Department of Psychosis Studies, Institute of Psychiatry, Psychology and Neuroscience, London, United Kingdom; 9 Kent and Medway Medical School, Canterbury, United Kingdom; 10 Kent and Medway National Health Service and Social Care Partnership Trust, Kent, United Kingdom

**Keywords:** sleep assessment, sleep quality, RDoC, compulsivity, neuroticism, anxiety, depression, mental health

## Abstract

Researchers are increasingly studying cognitive and psychological constructs using automated online tools due to advantages in scalability, repeatability, accessibility and affordability. The online assessment of sleep presents a challenge, as the most popular instruments for reporting different aspects of sleep were originally designed to be deployed under supervised conditions by trained personnel. Here, we develop and validate the Comprehensive Online Sleep Monitoring Scale (COSMOS), a self-reported sleep scale optimised for online, independent administration. Using data from *N* = 5,815 adults, we show that COSMOS has good internal (Cronbach’s *α* = 0.85) and convergent validity, via strong associations with established sleep scales. Poorer COSMOS sleep scores were found in participants diagnosed with mental health conditions, more frequent depression or anxiety symptoms, and higher compulsivity or neuroticism traits, demonstrating good construct validity. We propose COSMOS as a comprehensive and validated sleep assessment that is suitable for large-scale online transdiagnostic and mental health research.

## Introduction

Sleep is a fundamental physiological process that plays an integral role in maintaining health and wellbeing (Foster, 2020; Ramar et al., 2021; Watson et al., 2015). Contrary to the conventional view of sleep problems being secondary symptoms of other conditions, emerging studies highlight that they can be associated with subsequent neuropsychiatric and neurological diagnoses (Freeman et al., 2020). For instance, Rapid Eye Movement Behaviour Disorder (RBD) is strongly associated with later emergence of neurodegenerative disorders including Parkinson’s disease and Dementia with Lewy Bodies (increased risk of around 90%; Galbiati et al., 2019). In psychotic, major depressive, and anxiety disorders, sleep disturbances are commonplace and may, in some cases, also predate the onset of new episodes (Ferrarelli, 2020; Hertenstein et al., 2023; Waite et al., 2020). The relevance of sleep extends beyond clinical conditions to the general population, with sleep consistency and morning preference being linked to better academic performance (Hershner, 2020), whereas shorter sleep durations and elevated disturbances are associated with risk of injury at work and lower productivity. Sleep disruption also greatly impacts memory consolidation and learning processes (Ashton and Cairney, 2021; Gutiérrez et al., 2024; Guttesen et al., 2023). Consequently, the examination of sleep has become widespread in psychological, psychiatric and neurological studies (Kucharczyk et al., 2012).

Sleep is complex, multistage and can be disrupted in a variety of ways. Studying sleep often necessitates the use of multiple measurement instruments designed to capture different relevant constructs. Critically, some of the most common scales were originally designed for deployment under supervised conditions by trained personnel or those with clinical expertise (Buysse et al., 1989; John et al., 1991; Klingman et al., 2017). This presents two key limitations to their utility. First, standard scales may not be ideal for deployment by non-sleep specialists engaged in research (Klingman et al., 2017). More importantly, there is a growing application of assessment scales online via peoples’ home devices – e.g., personal computers, tablets, and smartphones. This shift to online deployment has been motivated by the scalability, repeatability, accessibility and affordability relative to supervised assessment methods, presenting an opportunity to study variability in neurological, psychiatric and mental health conditions in previously unachievable population size and detail (Bălăeţ et al., 2024; Binoy et al., 2024; Hampshire et al., 2020). However, a barrier to realising this potential is that scales designed for supervised deployment can produce different score distributions when deployed online (Morton et al., 2020); therefore, they require either online validation and recalibration or specific development taking into consideration the online medium they are to be deployed in.

The ubiquity of digital technologies also has direct relevance to sleep problems; specifically, the dynamics of sleep-related behaviours have been evolving in response to the increasing centrality of technology in our daily lives. Extensive use of personal and home technological devices such as mobile phones, tablets, televisions, and e-readers can substantially affect sleep behaviours and the quality of sleep one experiences (Liu et al., 2019; Scott and Woods, 2019). Specifically, cumulative evening screen use has been associated with greater sleep inertia (i.e., time in bed before getting up) and latency (i.e., time in bed before falling asleep), greater daytime dysfunction, poorer subjective sleep quality, and greater number of sleep disturbances (Castro-Santos et al., 2023; Šmotek et al., 2020). The exposure to bright light, particularly blue light, that accompanies screen use can be linked to these sleep disruptions through multiple mechanism. This is not only by the suppression of melatonin secretion and delaying of circadian phase (Wahl et al., 2019; West et al., 2011), but also by violating the basic principles of sleep hygiene by increasing pre-sleep cognitive, emotional, and physiological arousal (Chow, 2022; Roy et al., 2026). Therefore, current sleep assessments must adapt not only for deployment through these technologies but also to measure their influences on sleep.

Here, we developed a novel and comprehensive questionnaire designed to be suitable for modern online assessment of sleep. The questionnaire targets constructs from popular sleep scales and extends beyond those constructs with unique items that specifically address sleep-related behaviours resulting from the accessibility of technological devices. Crucially, we specifically tailored the wording of items to be unambiguous when deployed under unsupervised conditions, then validated them through deployment on an internet- and app-based research platform (Cognitron).

We intended that this Comprehensive Online Sleep Monitoring Scale (COSMOS), would measure eight key facets of sleep. These included (1) sleep disturbance, (2) daytime dysfunction, (3) symptoms indicative of sleep disorders, (4) bedtime routines, which included extent of screen time before bed, (5) medication use to support sleep, (6) chronotype, or natural preference to be active in the morning or evening, (7) sleep latency and duration, and (8) sleep quality. We expected COSMOS to show good, convergent validity when compared to established and widely used scales including the Pittsburgh Sleep Quality Index (PSQI)(Buysse et al., 1989), Morningness-Eveningness Questionnaire (MEQ)(Horne and Ostberg, 1976), and Epworth Sleepiness Scale (ESS) (John et al., 1991).

Given the intended application of this sleep scale in future psychiatry research, and to investigate the scale’s construct validity, we explored the relationship between COSMOS and psychiatric conditions, psychological symptoms and risk-associated traits previously linked with sleep disruptions (Freeman et al., 2020). We hypothesised that worse sleep scores, as measured by COSMOS, would be evident in people reporting one or more pre-existing mental health diagnoses and those with elevated frequencies of depression or anxiety symptoms as measured using items from the nine-item Patient Health Questionnaire (PHQ-9); (Kroenke et al., 2001) and Generalized Anxiety Disorder-7 (GAD-7) (Spitzer et al., 2006), since sleep disturbances are common symptoms of both conditions. Compulsivity is characteristic of several psychiatric disorders, particularly obsessive-compulsive-related disorders, in which sleep disturbances are prevalent (Segalàs et al., 2021; Tiego et al., 2023). We expect that higher compulsivity as measured by the Cambridge–Chicago Compulsivity Trait Scale (CHI-T) (Chamberlain and Grant, 2018; Tiego et al., 2023) will be associated with higher scores on COSMOS. We also sought to assess the relationship of COSMOS measures with neuroticism as measured by an abbreviated version of the Big Five Personality Inventory (Gosling et al., 2003; John et al., 1991), as this personality trait has been proposed as a potential risk factor across psychiatric conditions (Kotov et al., 2010; Lahey, 2009; Ormel et al., 2013; Schirmbeck et al., 2015) and has been identified as an independent risk factor for sleep problems (Akram et al., 2023). We benchmarked the discriminative sensitivity of COSMOS relative to established sleep scales when assessing their associations with the above conditions, symptoms, and traits.

## Methods

### Participants, design, and procedure

Twenty-eight thousand seven-hundred fifty-two (28,752) participants who had taken part in a follow-up cognitive assessment at the beginning of 2022 as part of the Great British Intelligence Test (GBIT; Hampshire, 2020) on the Cognitron testing platform were invited to this study. The study was approved by Imperial College London ethics (REF: 17IC4009) and participants provided informed consent online before completing the tasks. Five-thousand eight-hundred fifteen complete responses were recorded. These responses were anonymized and follow-up IDs were removed during data extraction to ensure only the minimal data necessary was shared. For full demographics see Table 1.

**Table 1:** Demographics.

Demographic variable	Description	N	%
		5,815	100.00
Gender	Total	4,808	82.68
	Male	1869	32.14
	Female	2,910	50.04
	Other	29	0.50
Age (Decade)	Total	5,214	89.68
	16–19	131	2.25
	20–29	489	8.41
	30–39	643	11.06
	40–49	844	14.51
	50–59	1,329	22.85
	60–69	1,339	23.03
	70–69	417	7.17
	80+	23	0.40
	Mean	51.78	
	SD	15.32	
Ethnicity	Total	5,236	90.04
	White	4,996	85.92
	Black	21	0.36
	Asian	135	2.32
	Mixed	79	1.36
	Other	5	0.09
Education level	Total	5,236	90.04
	pre-GCSE	65	1.12
	School	1,471	25.30
	Degree	3,409	58.62
	PhD	291	5.00
First language	Total	4,808	82.68
	English	4,564	78.49
	Other	244	4.20
Employment status	Total	5,237	90.06
	Worker	2,951	50.75
	Retired	1,629	28.01
	Student	292	5.02
	Unemployed	138	2.37
	Homemaker	145	2.49
	Other	82	1.41

COSMOS, PSQI, ESS, and MEQ were presented to all participants in this fixed order, with visible scale titles. Participants could complete the questionnaire on their personal devices (e.g., mobile phone, tablet, laptop, personal computer) and browser of choice.

### Materials

#### Sleep metrics

COSMOS Development. A non-exhaustive list of common sleep assessment scales was compiled based on a literature search in PubMed using the following terms: (sleep quality[Title]) AND ((psychometric*[Title]) OR (questionnaire[Title]) OR (scale[Title]) OR (index[Title]) OR (diagnos*[Title]) OR (instrument[Title])). After filtering for studies conducted with human samples, the number of search results was narrowed down to 194 from 241.

The papers’ relevance was evaluated based on their titles and abstracts, noting the scales used or mentioned. Of the eighteen unique scales that were identified, thirteen were deemed relevant based on a deeper inspection of the literature. Scales that were sample- or disorder-specific were removed.

Items from the thirteen scales carried forward were evaluated to identify common themes and items (see Supplementary Appendix A for full list of scales). Items common across multiple scales were collapsed and the frequency of the item was noted. The items were then grouped based on these broad themes and further categorized based on the specific subject of the item. For example, item variants on the subject “Do you snore (loudly)?” were found in seven of the scales and classified as “breathing issues” and then subcategorized under “snoring.” This narrowed down the questions from 334 to 74 items.

A shortlist of relevant subjects and questions to include was created based on the number of items in each category, how common items were across the scales, and the relevance of the items to assessments of sleep in the general population. This yielded 49 items of which 10 were removed due to having too much overlap in content. A list of 31 essential items was created and reworded to fit with the intention of unsupervised online administration and response within the web browser.

**Figure 1:**
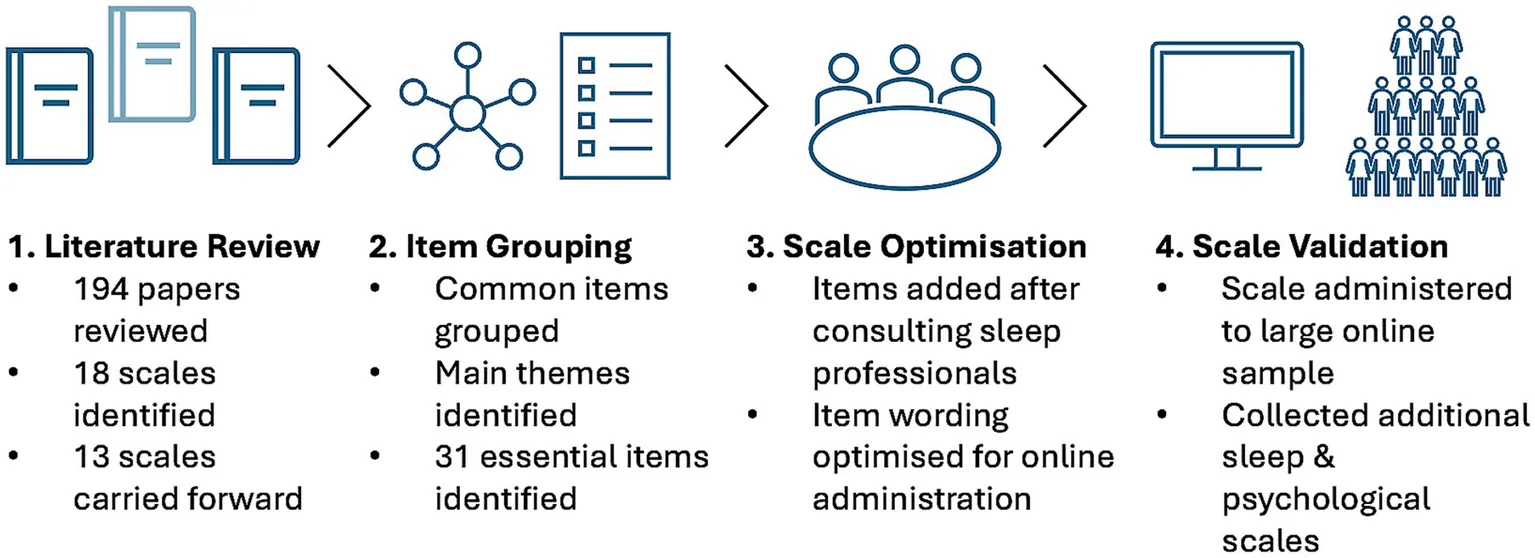
Scale development process. An overview of the literature review, evaluation and optimization of scale items, and the scale validation processes.

After consultation with researchers and sleep experts, items were added, removed, and modified. A “bedtime habits” subscale was added to capture common behaviours around sleep primarily covering technology use, reading, and prayer or meditation. More detail around drug or medication use to support sleep was added to encompass illegal drugs, cigarette smoking, and alcohol consumption as well as prescribed and over-the-counter medications. These were phrased in the context of use to aid sleep rather than general consumption. Questions about the quantity of use were added. Additional sleep onset and wake-up items were added to capture any disparity in sleep duration and onset from weekdays to weekends, also known as social jetlag. Finally, three free text, or open-ended, items were added to allow participants to share, in their own words, details about any medical conditions that may affect sleep quality, sleep problems experienced in the past four weeks, and if their sleep over the past four weeks differed from the past few years and why (see Figure 1 for a summary of the scale and study design).

The final scale (Table 2) comprised a total of 49 compulsory questions that were displayed to every participant and 17 nested items that were only completed conditional on the response to the previous question. For instance, participants who indicated they never took naps did not receive questions about the frequency, duration, and spontaneity of their naps.

**Table 2:** COSMOS items and response scale.

	Item	Response	Dependency	Subscale
The following questions ask about your sleep quality and habits. Please answer based on the past 4 weeks.				
How often did you:				
1	Find it difficult to fall asleep at night	Default		Latency
2	Find it difficult to stay asleep at night	Default		Disturbance
3	Find it difficult to fall asleep because your mind felt like it was racing with thoughts or worries	Default		Disturbance
4	Awaken with racing thoughts or worries	Default		Disturbance
5	Have trouble sleeping at night due to your room being too hot or cold	Default		Disturbance
6	Have trouble sleeping at night due to your environment being too noisy	Default		Disturbance
7	Have trouble sleeping at night due to your own coughing, snoring, or difficulty breathing	Default		Disorder
8	Have trouble sleeping due to other discomfort such as aches, pains or itching	Default		Disorder
9	Frequently wake up during the night	Default		Disturbance
10	Find it difficult to fall asleep after waking up during the night	Default		Disturbance
11	Wake up earlier than planned and were not able not get back to sleep	Default		Disturbance
12	Go to sleep and wake up at the same time every day	Default		Chronotype
13	Have very deep sleep	Default		Quality
14	Wake up feeling energized and rested	Default		Quality
15	Feel tired and fatigued during the day	Default		Dysfunction
16	Take naps during the day	Default		Dysfunction
17	Were your naps scheduled?	Default	Response to 16 is not never	Dysfunction
18	What was your average nap duration? [X minutes]	Minutes	Response to 16 is not never	Dysfunction
19	Find it difficult to fulfil work and everyday responsibilities due to poor sleep	Default		Dysfunction
20	Feel sleepy during the day which made it difficult to stay awake during everyday activities	Default		Dysfunction
21	Feel that your sleep was disturbed due to moving or speaking during your sleep	Default		Disturbance
22	Sleepwalk	Default		Disorder
23	Act out your dreams	Default		Disorder
24	Did this disturb your partner/members of your household?	Default	Response to 23 is not never	Disorder
25	Grind your teeth or clench your jaw during sleep	Default		Disorder
26	Have leg twitching or jerking during sleep	Default		Disorder
27	Have trouble sleeping or wake up at night because of intense nightmares	Default		Disorder
28	Use a phone or laptop before going to sleep	Default		Bedtime habits
29	Did you use a dark screen / night mode?	Default	Response to 28 is not never	Bedtime habits
30	Feel compelled to check your email, social media, or news after going to bed	Default		Bedtime habits
31	Did you try to stop yourself but failed?	Default	Response to 30 is not never	Bedtime habits
32	Did you check work related content (e.g., emails, work communication channels)?	Default	Response to 30 is not never	Bedtime habits
33	Did you check social media (e.g., Facebook, Instagram)?	Default	Response to 30 is not never	Bedtime habits
34	Watch videos before going to sleep (e.g., television, YouTube, TikTok)	Default		Bedtime habits
35	Read before going to sleep	Default		Bedtime habits
36	Did you read physical or electronic copies of books?	Physical/electronic/both	Response to 35 is not never	Bedtime habits
37	Did you use a dark screen / night mode?	Default	Response to 36 is electronic or both	Bedtime habits
38	Give up trying to sleep and read instead	Default		Bedtime habits
39	Give up trying to sleep and used your phone, laptop, or television instead	Default		Bedtime habits
40	Meditate or pray before going to sleep	Default		Bedtime habits
How often did you do the following to help fall or stay asleep?				
41	Drink alcohol in the evening	Default		Substance
42	How much did you drink?	Units [Numeric entry]	Response to 41 is not Never	Substance
43	Smoke cigarettes	Default		Substance
44	How many cigarettes did you smoke?	Numeric entry	Response to 43 is not Never	Substance
45	Take prescription drugs	Default		Substance
46	Please list the name(s) and dose(s).	Free text	Response to 45 is not Never	Substance
47	Take over the counter or herbal drugs	Default		Substance
48	Please list the name(s) and dose(s).	Free text	Response to 47 is not Never	Substance
49	Take illegal drugs	Default		Substance
50	Please list the name(s) and dose(s).	Free text	Response to 49 is not Never	Substance
51	I go to bed at (this may be different to the time you fell asleep) [X].	24-Hour (15-Minute intervals)		Sleep Onset
52	Once I’m in bed, I start trying to fall asleep after [X minutes].	Minutes (Numeric entry)		Sleep Onset
53	Once I decide to sleep, falling asleep takes me [X hours].	Hours (Numeric entry)		Latency
54	I sleep for [X hours].	Hours (Numeric entry)		Duration
55	My sleep schedule differs from weekdays to weekends.	Yes/no		Social Jetlag
56	On weekdays, I go to bed at [X] and wake up at [X].	24-Hour (15-Minute intervals)	Response to 55 is Yes	Social Jetlag
57	On weekends, I go to bed at [X] and wake up at [X].	24-Hour (15-Minute intervals)	Response to 55 is Yes	Social Jetlag
58	I wake up at [X]	24-Hour (15-Minute intervals)	Response to 55 is No	Social Jetlag
59	For me, a good night’s sleep lasts [X hours].	Hours (Numeric entry)		Duration
60	I naturally prefer to sleep and wake up early.	0–4; Strongly disagree/disagree/neither agree nor disagree/agree/strongly agree		Chronotype
61	I normally wake up [X] times during the night when trying to sleep.	Numeric entry		Disturbance
62	I would rate the quality of my sleep as	0–4; Very poor/poor/average/good/very good		Quality
63	I share a bed or room.	Default		Disturbance
64	I have a medical condition that makes it difficult for me to get good quality sleep.	Yes/no		Disturbance
65	Please describe	Free text	Response to 63 is Yes	
66	Please describe in your own words the nature of any sleep problems that you have had over the past four weeks.	Free text		
67	Please describe in your own words if, how, and why your sleep over the past four weeks differs to the past few years.	Free text		

Default response: 0–5; never/almost never/once or twice a week/several times a week/nearly every day/every day.

The questionnaire specified that responses should be based on the past four weeks. The majority of items were scored on a six-point Likert scale from zero, or “never,” to five, “every day.” Exceptions to this included some nested items, where a more specific response was appropriate (e.g., yes / no questions, quantities and dosage, and screen time specifications), timings such as sleep onset or duration, free text items, a sleep quality item that asked for a rating of sleep quality on a five-point Likert scale ranging from “very poor” to “very good,” and a propensity to morning chronotype item which was rated on a five-point Likert scale ranging from “strongly disagree” to “strongly agree.”

PSQI. The PSQI (Buysse et al., 1989) was used to obtain a measure of sleep quality and its components more generally. This 19-item scale generates a global sleep quality metric alongside scores for seven components of sleep quality including, self-rated sleep quality, sleep latency, sleep duration, sleep efficiency, sleep disturbances, daytime dysfunction, and medication use around sleep. The scores for each component range from zero to three and these scores are then added, resulting in a single global score from 0–21 with higher scores indicating poorer sleep quality. The scale has been used widely for research and has good reliability, with a Cronbach’s (Cronbach, 1951) alpha (*α*) of 0.75 in the current sample.

MEQ. Chronotype, or circadian rhythm preference, was measured using the reduced MEQ (Horne and Ostberg, 1976). Here the respondent is asked to indicate the times they feel most alert or tired as well as their sleep–wake preferences. The questionnaire consisted of five items and responses were recorded on a Likert scale. Scores were calculated based on the total score for each item after reverse coding respective items such that higher scores indicated morning-type groups and lower scores evening-type individuals. The scale is widely used and reliable; Cronbach’s *α* of 0.73 in the current sample.

ESS. To assess daytime dysfunction - or sleepiness - we utilized the ESS (John et al., 1991). The ESS measures subjective sleepiness, Cronbach’s *α* of 0.82 in the current sample. The scale presents eight everyday situations where the respondent is asked to rate their likelihood to doze off on a four-point Likert scale from “no chance of dozing” to “high chance of dozing.” Responses are added to produce a total score from 0 to 24, where higher scores indicate greater daytime sleepiness.

#### Mental health diagnoses

The presence of a neurological or psychiatric illness was assessed through self-report. If respondents indicated they had been diagnosed with a neurological condition, psychiatric condition, or both, they were provided with a list of relevant diagnostic labels to select from. For psychiatric disorders, respondents could choose from Depression, Anxiety, Attention Deficit Hyperactivity Disorder, Obsessive Compulsive Disorder, Bipolar Disorder, or other. Multiple options could be selected at once. Respondents that indicated they did not have any neurological or psychiatric diagnoses were not presented with these two follow-up questions.

#### Depression and anxiety symptoms

Depressive and anxious symptoms were measured using a subset of five items from the PHQ-9 (Kroenke et al., 2001) and full GAD-7 (Spitzer et al., 2006). To ensure suitability for the general population and due to ethical constraints at the time of data collection, specific items from the PHQ-9 related to appetite, self-worth, agitation, and suicidality were excluded. Both scales were scored on a seven-point Likert scale ranging from 0–6 (“Never,” “Almost Never,” “Once or Twice a Week,” “Several Times a Week,” “Daily,” “Hourly,” More Often”) rather than the original response scales to allow for greater response variability and precision in a general population sample. Higher scores indicated poorer mental health. The scales had good internal consistency based on the current sample, PHQ Cronbach’s 𝛂 = 0.86, GAD-7 𝛂 = 0.92.

#### Compulsivity and big five personality traits

The CHI-T (Chamberlain and Grant, 2018), a short comprehensive compulsivity metric, was utilised to capture compulsive traits. The 15-item scale has two latent factors, one relating more to perfectionism and the second to compulsive soothing behaviours (Tiego et al., 2023). Some studies have also reported a three factor model, with perfectionism, cognitive rigidity, and reward drive, where cognitive rigidity and reward drive are included here under compulsive soothing (Hampshire et al., 2021). Higher scores indicated greater compulsive traits. The scale had good internal consistency, with a Cronbach’s 𝛂 of 0.80 based on this current sample.

An abbreviated 18-item version of the Big Five Inventory (John et al., 1991; Kang et al., 2024) was administered to capture personality traits, primarily neuroticism. The scale has five established factors – openness, agreeableness, extraversion, conscientiousness, and neuroticism – where the combination of scores on each describes an individual’s personality traits. Higher scores indicated greater alignment with a given trait. The scale had good internal consistency, with a Cronbach’s 𝛂 of 0.73 in the current sample.

### Analysis

#### Tools and software

Confirmatory factor analyses were conducted in R version 4.2 (R Core Team, 2021) using the Lavaan package (Rosseel, 2012). All other data processing and analyses were conducted using Pandas in Python version 3.9 (The Pandas Development Team, 2022). Pingouin (Vallat, 2018) was used to run ANOVAs and respective *post hoc* tests, Statsmodels (Seabold and Perktold, 2010) to test multiple regression models, Scipy (Virtanen et al., 2020) to generate correlations and associated *p*-values, and FactorAnalyzer (Biggs, 2022) to create fitted exploratory factor models from which individual factor scores were extracted.

#### Pre-processing

All items were converted from string responses to Boolean or scaled responses. Items were appropriately reversed such that all higher scores were indicative of poorer sleep behaviour or quality. Any missing data or skipped questions were coded as “not a number” (“NaN”). Free text items were dropped from further analyses described here. No imputation of missing variables was applied in the analyses. Scores were all z-scored.

#### Scoring

COSMOS. Social jetlag was calculated by taking the difference in sleep duration on weekends from weekdays. These sleep durations were calculated using the weekday and weekend bedtime and wake-up time items (items 56–59, Table 2). Those who indicated no difference in their sleep during the week and weekend received a score of zero. These items were then dropped from further analyses for all participants.

Depression and Anxiety Symptom Scales. A total score was calculated for depressive symptoms by taking the sum of the subset of PHQ items administered. The same was done using the full GAD-7 to calculate a total score for symptoms of anxiety.

Compulsivity and Neuroticism Scales. To calculate scores for the CHI-T compulsivity factor, exploratory factor analysis (EFA) was used to extract factor scores for the two CHI-T factors, as per Tiego and colleagues (Tiego et al., 2023). Factors were extracted using principal axis factoring and a varimax rotation was applied. Individual scores for each factor were then calculated using the fitted factor model. The compulsive soothing factor was taken forward for analysis rather than perfectionism as the items under the compulsive soothing but not perfectionism factor has been associated with poorer functional outcomes (Hampshire et al., 2021) (see Supplementary Appendix B for full EFA results).

Principal axis factoring set to five factors with varimax rotation was applied to the Big Five Inventory to extract factor scores. Neuroticism scores were multiplied by −1 such that higher scores indicated higher levels of neuroticism and then were carried forward for analysis (see Supplementary Appendix C for full EFA results).

#### Model fit parameters

Model fit parameters were interpreted based on standard cutoffs reported in the literature. Comparative fit indices (CFI) greater than 0.96 were interpreted as “good” (Hu and Bentler, 1999) and root mean square error of approximation (RMSEA) values less than or equal to 0.05 were considered indicators of close model fit and reasonable fit if between 0.05 exclusive and 0.08 inclusive (Byrne, 1994).

## Results

### Internal structure

The full item COSMOS showed good internal consistency as indicated by a Cronbach’s *α* of 0.85 (see Supplementary Appendices D, E for subscale-level Cronbach’s *α*). A confirmatory factor analysis (CFA) was conducted to test the hypothesized eight-factor structure of COSMOS, using full information maximum likelihood estimation to utilize all available information rather than excluding cases listwise. Nested variables were defined in the model as additional covariances. The model showed reasonable fit, *χ^2^*(1666) = 31026.86, *p* < 0.001, CFI = 0.81, RMSEA = 0.06. See Supplementary Appendix D for full factor loadings.

### Convergent validity

A multistep approach was taken to assess the extent to which COSMOS aligned with established sleep questionnaires. First, a second CFA of COSMOS was conducted using the hypothesized factor structure but separating the sleep latency and duration factor into four distinct factors: sleep latency, sleep duration, sleep onset, and social jetlag. This was done to make the COSMOS factor structure more similar to the structure used in the established scales. The model showed a slightly poorer fit to the original eight-factor model, *χ^2^*(1368) = 25089.80, *p* < 0.001, CFI = 0.78, RMSEA = 0.06. See Supplementary Appendix E for full factor loadings.

Next, a CFA of the established scales was conducted using a nine-factor model. These were made up of the seven predefined PSQI factors, daytime dysfunction which included the ESS items, and a chronotype factor containing the MEQ items. The model showed a similar fit quality to COSMOS models, *χ^2^*(399) = 9274.80, *p* < 0.001, CFI = 0.80, RMSEA = 0.06. See Supplementary Appendix F for full factor loadings.

**Figure 2:**
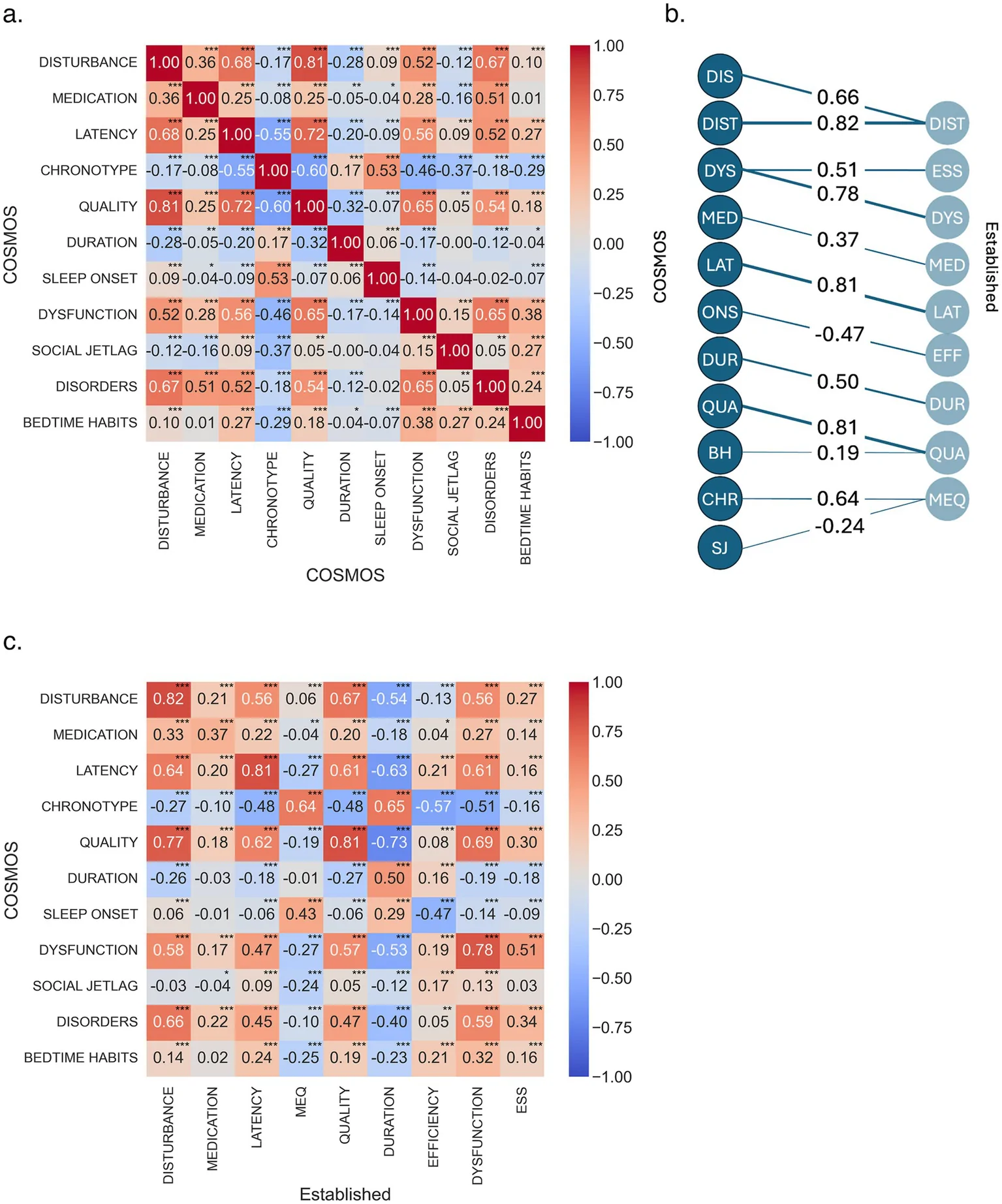
Cosmos latent variable score correlations. **(a)** COSMOS latent variable model subscale score correlations and **(b)** corresponding variable correlations between COSMOS (left) and the established scale latent variable models (right). **(c)** An asymmetric correlation matrix describing the correlations between corresponding scales across COSMOS and the established model. *p*-values all corrected for multiple comparisons using Bonferroni correction, ***p < 0.001; **p < 0.01; *p < 0.05.

These fitted models of COSMOS and established scales were used to extract individual latent variable scores for each of the factors. A Pearson correlation with a Bonferroni correction for multiple comparisons was calculated for each pair of scores to assess the relationship between the subscales (Figure 2). This showed statistically significant correlations of moderate to strong effect size between the COSMOS subscales and the corresponding established subscales. The correlation between medication subscales was statistically significant but weak.

The social jetlag, disorders, and bedtime habit subscales did not have clear corresponding established subscales to be compared to in this study. Social jetlag scores did not correlate well with any of the subscales but showed a significantly weak negative correlation to the MEQ. Similarly, the bedtime habits subscale was weakly but significantly correlated with timing-related subscales including the MEQ, latency, and efficiency and the dysfunction subscale.

### Associations

#### Self-report psychiatric diagnostic labels

A mixed ANOVA was conducted to assess sleep differences across the COSMOS subscales between participants who had no psychiatric conditions (*n* = 5,024) and those who reported having at least one psychiatric, but no neurological conditions (*n* = 506). Participants who indicated having a neurological condition were excluded from this analysis (*n* = 285). Permutation-based-*p*-values (*n* = 5,000) were also calculated to account for any violated normality assumptions. See Table 3 for included disorders.

**Table 3:** Number of participants in each diagnostic group.

Diagnosis	Count
None	5,024
Depression	330
Anxiety	89
Obsessive compulsive disorder	36
Other	32
Attention deficit hyperactivity disorder	19

**Figure 3:**
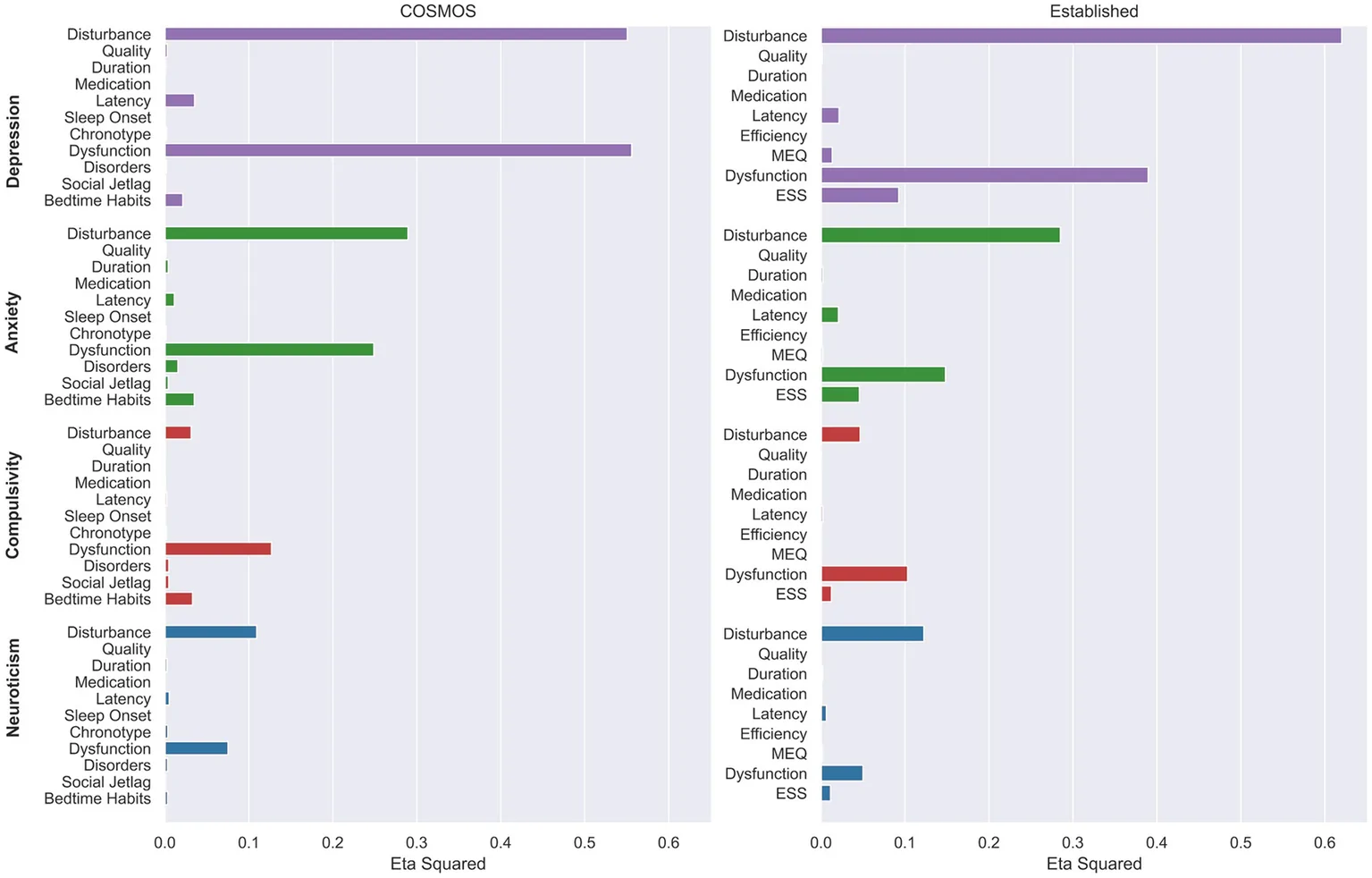
Difference in COSMOS subscale latent variable scores between participants diagnosed with any or no psychiatric condition. *Post-hoc* test of z-scored latent variable scores for each COSMOS subscale with Bonferroni correction for multiple comparisons, ***p < 0.001, **p < 0.01; *p < 0.05.

There was a significant main effect of self-reported psychiatric diagnosis on total COSMOS scores, *F* (1, 5,528) = 250.46, *p* < 0.001, *η*_p_^2^ = 0.043, *p*-perm < 0.001. Pairwise post-hoc tests were conducted with a Bonferroni correction of multiple comparisons. These showed significantly higher scores on all COSMOS subscales, except for the Chronotype subscale where this effect was reversed (Table 4). The mean differences were significant for every subscale except duration (Figure 3). The effect sizes ranged from small to large, with the dysfunction subscale showing the largest effect.

**Table 4:** Mixed ANOVA post-hoc results subscales x psych group with FDR corrected *p*-values and Cohen’s *d*.

Subscale	*t* (df)	*p*	d
Disturbance	−7.55 (590.8)	<0.001	−0.38
Dysfunction	−15.66 (572.34)	<0.001	−0.87
Disorders	−11.89 (572.47)	<0.001	−0.66
Bedtime habits	−8.12 (589.18)	<0.001	−0.42
Medication	−7.75 (574.43)	<0.001	−0.43
Latency	−11.5 (580.91)	<0.001	−0.61
Duration	−0.94 (574.08)	0.349	−0.05
Sleep onset	2.04 (600.95)	0.046	0.10
Social jetlag	−2.98 (610.45)	0.004	−0.14
Chronotype	10.1 (598.11)	<0.001	0.50
Quality	−11.35 (605.04)	<0.001	−0.54

#### Depression and anxiety symptoms

The relationships between COSMOS and established anxiety and depression symptom scales were assessed using multiple linear regressions. Separate multiple linear regression models were run with COSMOS latent variable scores as the independent variables and either the depression or anxiety scores as the dependent variable. The same analyses were conducted using the latent variables extracted from the established scales’ CFA as the independent variables. Full results can be found in Supplementary Appendix G.

**Figure 4:**
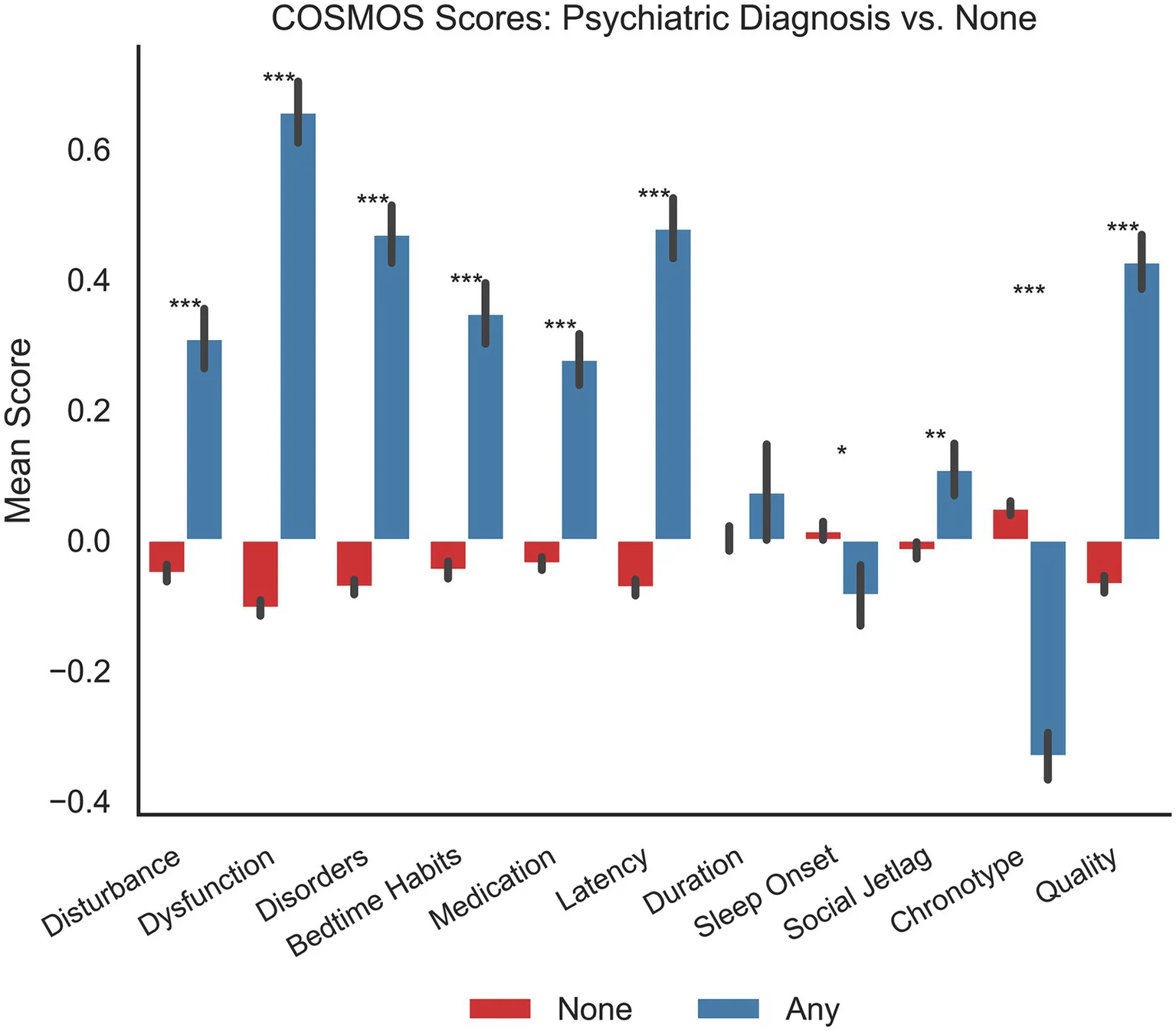
Effect sizes of sleep subscales in relation to psychiatric symptoms and traits. Eta squared of regression coefficients for COSMOS and established scale subscales when estimating psychological symptoms and traits.

The COSMOS and established scale models were both statistically significant and explained over half of the variance in depression scores [*F*(11, 5,225) = 556.80, *p* < 0.001, *p-perm* < 0.001, *R*^2^ = 0.54; *F*(9, 5,227) = 661.70, *p* < 0.001, *p-perm* < 0.001, *R*^2^ = 0.53, respectively]. The COSMOS dysfunction subscale showed a large effect size followed by small effect sizes for the bedtime habits and latency subscales (Figure 4).

COSMOS and established scales both significantly explained over 30% of the variance in anxiety scores, with COSMOS explaining 38% [*F*(9, 5,227) = 290.80, *p* < 0.001, *p-perm* < 0.001, *R*^2^ = 0.38] and established scales 34% [*F*(9, 5,227) = 293.60, *p* < 0.001, *p-perm* < 0.001, *R*^2^ = 0.34] of the variance. Again, the dysfunction subscale contributed most to increases in GAD scores as shown by its large effect size along with disorders, bedtime habits and latency which had small effect sizes (Figure 4).

#### Personality traits

Separate multiple linear regression analyses were conducted between the COSMOS subscales and extracted CHI-T compulsivity and BIG-5 neuroticism factors. Identical analyses were conducted with the established scales’ factor scores as the independent variable. COSMOS significantly predicted scores on the compulsive soothing factor, explaining 18% of the variance [*F*(11, 5,225) = 98.31, *p* < 0.001, *p-perm* < 0.001, *R*^2^ = 0.18]. This was similar for the established scales, [*F*(9, 5,227) = 95.97, *p* < 0.001, *p-perm* < 0.001, *R*^2^ = 0.14]. COSMOS’s dysfunction subscale had the greatest contribution, with a medium effect size (Figure 4). The bedtime habit and social jetlag subscales’ contributions had small effect sizes (Figure 4).

The model using COSMOS subscales to predict scores on the extracted neuroticism factor was statistically significant [*F*(11, 5,224) = 97.28, *p* < 0.001, *p-perm* < 0.001, *R*^2^ = 0.17] and this was very similar to that of the established scales [*F*(9, 5,226) = 113.10, *p* < 0.001, *p-perm* < 0.001, *R*^2^ = 0.16]. The dysfunction subscale, again, contributed most to this, with a medium effect size (Figure 4). See Supplementary Appendix G for the full set of results. Repeating the analyses but with independent models for each predictor produced the same pattern of results but with larger effect sizes (Supplementary Appendix H), likely due to feature collinearity (see Supplementary Appendix G for VIF and tolerance values).

## Discussion

There is great potential utility in being able to assess sleep quality and its components comprehensively and remotely without supervision, including deployment longitudinally at a large population scale. COSMOS was developed to address these needs and, here, we demonstrate that it has good convergent validity with popular established scales and equivalent or stronger associations to psychiatric conditions, mental health symptoms and personality traits.

When applying a confirmatory approach to examine the internal structure of COSMOS, where items were assigned to factors according to the extant sleep literature, and taking inspiration from the established scales used in this study, the model provided a comparable fit for the data as those established scales. Furthermore, COSMOS showed high internal consistency as measured by Cronbach’s alpha. Corresponding scores from COSMOS and established scales correlated well. Taken together, these results demonstrate good validity of the constructs COSMOS was intended to measure.

COSMOS was created to be a comprehensive tool for current times; therefore, it includes some elements not captured by the established PSQI, ESS, or MEQ scales. For example, medication in the PSQI includes over-the-counter or prescribed medication to support sleep whereas COSMOS does so for *any* drug or substance use in the context of aiding sleep, including alcohol, cigarettes, and illicit substances, over and beyond just over-the-counter and prescription medications. This added variance could explain the weaker correlation observed between these two subscales, particularly as some of the additional substances, such as alcohol, could impair sleep despite the intended use as a sleep aid (He et al., 2019). Another consideration is that this scale was administered in the United Kingdom where cannabis use remains illegal. Therefore, this information may be unintentionally categorised under “over-the-counter” or “prescription medication” if the scale was administered in a country where cannabis use was legal. For a more generalisable global scale, future iterations may benefit from explicitly addressing cannabis or listing it as an independent item.

The instrument showed good construct validity, with poorer COSMOS sleep scores in participants diagnosed with mental health conditions, more frequent depression or anxiety symptoms, and higher compulsivity or neuroticism traits. We evaluated the associations between COSMOS and specific aspects of mental disorders including the presence of any disorder as well as scores relating to depression and anxiety. Links between COSMOS and compulsivity, as well as Big Five personality traits were evaluated. The relevance of sleep in understanding psychiatric disorders has been well-established, a significance that grows as we move toward a transdiagnostic approach in psychiatric diagnosis and treatment (Freeman et al., 2020). Given this emerging perspective, we sought to create a scale suitable for assessing sleep quality while remaining attuned to trait-level variations in psychological symptoms. Transcending clinical boundaries enables us to characterise psychiatric risk factors and their relationship with sleep quality, contributing to a deeper understanding of defined psychiatric diagnoses. In line with this transdiagnostic view, we found that COSMOS scores were not only higher for participants with psychiatric diagnoses, with large effect sizes, but also of neuroticism and compulsivity traits, which are psychological risk factors, and recent symptoms of depression. Interestingly there was an overlap in the subscales which contributed most to these associations.

Daytime dysfunction due to sleepiness and bedtime habits such as screen time or reading were top contributors to each of these effects. This finding mirrors what has been reported in the literature about technology use and poor mental health outcomes (Demirci et al., 2015; Elhai et al., 2017). For anxiety and depression, we also observed that the impact of sleep latency and disturbance alongside both of these which is in concordance with symptoms commonly experienced in numerous psychiatric populations (Freeman et al., 2020; Johnson et al., 2006; Mason and Harvey, 2014). Not only does this support associations between our novel scale and psychological traits, but it also demonstrates the contributions of the additional bedtime habit items.

There was no direct corresponding comparison that could be made between the evaluated established scales and our more experimental subscales: bedtime habits and social jetlag. Nonetheless, these subscales were included in COSMOS as they captured information relevant to current sleep behaviour and lifestyle. The bedtime habits scale showed weak correlations with subscales relating to sleep timing and sleep dysfunction. These weak correlations are not entirely surprising as these subscales measure qualities adjacent to that of the established subscales rather than a perfect overlap in content. Social jetlag similarly showed a weak negative correlation to morningness-eveningness but no clear relationship to the other scales. This negative relationship is more surprising as greater differences in weekend and weekday sleep timings have been reported as typical of people with evening-type chronotypes (Roenneberg et al., 2012). It is important to note that several social jetlag calculations have been documented including taking the difference in sleep midpoints or wake times (Roenneberg et al., 2012). Here we calculated the difference in duration which captures differences in sleep length. However, it is possible that a social jetlag calculation that accounts for differences in actual timing or sleep midpoint would link more closely to a chronotype measure.

Both social jetlag and screen time around bedtime have been implicated as risk factors for mental health issues. Moreover, social jetlag has been associated with a greater risk of experiencing depressive symptoms (Levandovski et al., 2011), and sleep quality mediates this relationship between bedtime screen time and poor mental health outcomes (Alonzo et al., 2021). To fully assess the validity and utility of these subscales, it is imperative to evaluate their sensitivity to the measures of interest.

The demonstrated association of COSMOS to psychological traits and its convergence with popular established tools is promising. Our choice of a large, online community is fitting considering the growing emphasis on studying transdiagnostic markers in contemporary psychiatric research (Gillan et al., 2017, 2016). Furthermore, we have shown that COSMOS is suitable for unsupervised online administration, which is essential for researchers moving to large-scale community-based samples.

COSMOS was designed to be comprehensive to address the issue of having to use multiple sleep quality questionnaires to capture different relevant aspects. Although we have achieved this, a consideration is the need for short scales in some studies, where various constructs are collected in the same session. COSMOS is modular, being deployable in relevant sub-sections for different purposes. Future work should focus on exploring whether an abbreviated version, with fewer items per subscale, can be achieved. We identified some items that could be removed or rephrased in future iterations of the scale based on the observed responses and their variability or their sensitivity to other scales. Questions about drug, alcohol, cigarette, and medication quantity did not have the expected spread, with some reported quantities going beyond plausible ranges, albeit these being of low incidence. This highlights the importance of using filters and flags for non-compliant responses. In future versions, asking participants about shiftwork would be helpful when calculating and interpreting certain items such as duration, onset, and social jetlag. Some questions about duration and sleep timing, particularly wake-up time, could be reduced in future versions of the scale due to their sparsity and redundancy.

Although we endeavoured to test a community-based sample, it is important to note that our sample had biases, including toward older, White, and higher educated participants. Even healthy ageing comes with changes in sleep duration, latency, and number of nighttime awakenings (Lavoie et al., 2018; Mander et al., 2017). Similarly, lower socioeconomic status and educational attainment have been related to shorter sleep duration or sleeping too much and more frequent sleep complaints (Grandner et al., 2010; Lee et al., 2021). Our sample was comprised mainly of native English speakers, meaning further validation would be required to ensure the scale’s suitability for use with non-native English speakers.

Additionally, ethical constraints at the time of data collection necessitated the exclusion of four items from the PHQ-9, limiting direct comparability with studies using the full scale. Furthermore, this scale validation study also ran at the end of a longer study which introduces further completion biases. Therefore, generation of normative scores for COSMOS requires further data collection with random sampling. This would also allow for the exploration of the interaction of sleep with clinical labels, psychological traits, or psychiatric symptoms to predict the emergence or intensification of mental health episodes and states. Although COSMOS was developed for transdiagnostic research purposes where continuous scores are more informative than binary classification, population-level normative data would also help provide useful reference points for interpreting individual scores in applied research contexts.

It is important to note that the data presented here were collected at the beginning of 2022, a period when the effects of the coronavirus disease (COVID-19) pandemic remained significant. COVID-19 infections disrupt sleep and the effects can be prolonged, with recovered patients reporting persistent sleep disturbances and changes in mood and energy levels months after their initial positive test (Nalbandian et al., 2021). Furthermore, environmental changes and disruptions to routine such as continued remote working and isolation orders may have influenced people’s self-reported sleep behaviours (Limongi et al., 2023; Yuan et al., 2022). Finally, the measure of fit in the CFA was moderate for both COSMOS and established scales. We acknowledge that the established scales were not designed to be scored in this way, however, it has also been noted that the restrictions imposed on model parameters in CFA, as described by Marsh and colleagues (Marsh et al., 2014), which increases the likelihood of having moderate model fit even for well-established scales (Marsh et al., 2010). While CFA was used here to allow for a more hypothesis driven approach, equivalent models using exploratory structural equation modelling have been estimated and can be found in the supplementary material (Supplementary Appendix I).

The assessment of sleep is inherently complex due to its multifaceted nature. Recognising the significance of studying sleep as a transdiagnostic construct in relation to psychiatric disorders is essential as we move toward more diagnostic-label-agnostic methods of understanding, diagnosing, and treating psychiatric disorders. COSMOS presents a new, validated, and sensitive tool optimized for unsupervised administration to enable very large longitudinal studies as mental health research continues to transition to online deployment.

## Data Availability

The raw data supporting the conclusions of this article will be made available by the authors, without undue reservation.
